# Errors and Predictors of Confidence in Condom Use amongst Young Australians Attending a Music Festival

**DOI:** 10.1155/2016/6054870

**Published:** 2016-11-13

**Authors:** Karina M. Hall, Daniel G. Brieger, Sukhita H. De Silva, Benjamin F. Pfister, Daniel J. Youlden, Franklin John-Leader, Sabrina W. Pit

**Affiliations:** ^1^School of Medicine, Western Sydney University, Locked Bag 1797, Penrith, NSW 2751, Australia; ^2^University Centre for Rural Health, Western Sydney University, 61 Uralba Street, Lismore, NSW 2480, Australia; ^3^Harm Reduction and Health Promotion Programs, HIV and Related Programs (HARP), North Coast Public Health, Mid-North Coast Local Health District, P.O. Box 419, Lismore, NSW 2480, Australia

## Abstract

*Objectives*. To determine the confidence and ability to use condoms correctly and consistently and the predictors of confidence in young Australians attending a festival.* Methods*. 288 young people aged 18 to 29 attending a mixed-genre music festival completed a survey measuring demographics, self-reported confidence using condoms, ability to use condoms, and issues experienced when using condoms in the past 12 months.* Results*. Self-reported confidence using condoms was high (77%). Multivariate analyses showed confidence was associated with being male (*P* < 0.001) and having had five or more lifetime sexual partners (*P* = 0.038). Reading packet instructions was associated with increased condom use confidence (*P* = 0.011). Amongst participants who had used a condom in the last year, 37% had experienced the condom breaking and 48% had experienced the condom slipping off during intercourse and 51% when withdrawing the penis after sex.* Conclusion*. This population of young people are experiencing high rates of condom failures and are using them inconsistently or incorrectly, demonstrating the need to improve attitudes, behaviour, and knowledge about correct and consistent condom usage. There is a need to empower young Australians, particularly females, with knowledge and confidence in order to improve condom use self-efficacy.

## 1. Introduction

The male condom has long been hailed as one of the best available methods for the prevention of sexually transmitted infections (STIs), short of abstinence. Consistent condom use is associated with reduced transmission of human immunodeficiency virus (HIV) and urethral infection in men, herpes simplex virus 2 (HSV-2), syphilis and chlamydia in both genders, and reduced acquisition of gonorrhoea and trichomoniasis in women [1]. Nearly 80% of STI notifications occur in people aged 15–29 years [2], and rates of diagnosis are increasing in Australia [3]. Notifications of chlamydial and gonococcal infection have almost doubled in 20–24-year-old Australians in the last decade [3]. STIs have the potential to cause serious complications such as pelvic inflammatory disease, tubal infertility, ectopic pregnancy, and chronic pelvic pain in women and carry a significant risk of harm to the unborn child [4, 5].

Despite the recognised value of male condoms, consistent use amongst young people is low. Two studies in the United States found that, among university students, just 28.4–30.6% report using a condom with every new sexual partner [6, 7]. Similarly, De Visser et al. found that only 48.4% of Australian men aged 20–29 always used condoms with casual partners [8]. Reasons for condom nonuse are numerous; however one less extensively researched measure that has been associated with greater condom use is user confidence, or self-efficacy (a measure of the degree of confidence in one's motivation* and* ability to perform a particular behaviour) [9–12]. Those with greater self-efficacy are more likely to engage in an activity than those with less confidence in their ability [13]. Accordingly, both greater confidence [9] and perceived self-efficacy [14, 15] have been associated with greater condom use. Emerging literature has demonstrated that confidence in one's ability to use a condom is as important as attitudes toward condoms for promoting future condom use [10]. Hence, understanding user confidence and its determinants may aid the development of strategies to promote consistent and effective condom use.

The young Australian population who attend music festivals have been recognised as a clinically relevant target group for intervention strategies due to their higher risk of STIs [16, 17] and engagement in high risk sexual behaviours such as inconsistent condom use [17]. A small number of studies have examined the predictors of condom use self-efficacy in other groups [18–20]; however to our knowledge no research has been conducted investigating condom use confidence or its influence on sexual behaviours in this group of young Australians.

This study aims to determine (1) the level of confidence young Australians attending a music festival have in their ability to use condoms correctly and consistently and (2) the predictors of confidence in using condoms.

## 2. Methodology

### 2.1. Recruitment

A convenience sample of 18–29-year-olds was conducted at a mixed-genre music festival in New South Wales (NSW). Data collection took place in a sexual health stall situated in the campgrounds of the festival. The stall consisted of volunteers facilitating a variety of interactive activities designed to promote sexual health. Participants entered the stall of their own volition or were invited by volunteers. Participants were excluded if they reported their age as less than 18 years or were visibly inebriated. Researchers were trained by those who had completed Responsible Service of Alcohol (RSA) training. The RSA is a standard qualification required by those who wish to serve alcoholic beverages in Australia and includes training in the recognition of intoxicated people by appearance and behaviour. The procedure of the survey was explained to all participants and anonymity of survey responses ensured. Verbal consent was obtained prior to commencement.

### 2.2. Data Collection

Participants completed an 11-question paper-based survey in private. The survey collected basic demographic information and used interval scale questions to ascertain self-reported confidence and ease of performing specific condom skills required for correct application. Participants were asked to report the number of standard drinks consumed in the past 24 hours and number of previous sexual partners in order to examine the relationship between risk-taking behaviours and confidence. Number of standard drinks consumed in the past 24 hours was not used as a measure for current intoxication. Variables such as sexuality and number of standard drinks consumed in the past 24 hours were determined based on personal identification and self-report. Participants were asked to indicate the source of information used when learning to apply condoms in tick-box format, whereby multiple answers could be selected, for example, high school sex education, family, friends, and internet. The Correct Condom Use Self-Efficacy Scale (CCUSS) was modified to measure condom use confidence [21]. This is a seven-item list that addresses one's own perceived ability to use a condom. The statements required participants to convey their level of agreement on a 5-point, Likert-type item. For example, “How easy or difficult would it be for you to put a condom on correctly?” Table 2 lists the questions asked in the modified CCUSS. Interval scales were used to determine the frequency of condom errors or failures in the past 12 months. The survey was piloted (*n* = 18) amongst a sample of university students aged 20–25 years to maximise acceptability. Explicit permission was obtained from festival organisers to conduct the study. All participants were offered a brief education session following the completion of the survey. The Western Sydney University HREC approved the study (H9067).

### 2.3. Statistical Analyses

In total, 290 participants were recruited. The surveys of two participants were excluded from data analysis due to largely missing data or invalid responses to survey items. IBM® SPSS® Statistics (Version 22) software was used to conduct analyses. Two tailed statistical tests were used. Chi-square and Fisher's exact tests were used to determine whether demographics and risk behaviours were associated with confidence [22, 23]. Continuous data was not normally distributed; therefore the median and interquartile range were given and the nonparametric Mann-Whitney *U* test was used to determine the association between age, number of standard drinks in 24 hours, number of lifetime sexual partners, and confidence [22]. Significance was defined as a *P* value ≤ 0.05 for all tests. The influence of source of condom use education on confidence was evaluated by comparing those who had not learnt about condoms from a particular source (i.e., high school, packet instructions, and friends) to those who had. When performing logistic regression analyses, Likert-scale determined confidence levels were regrouped into either “confident” or “not confident.” The “confident” group consisted of those who reported themselves as “extremely” or “mostly confident” in using condoms, whilst the “not confident” group consisted of those who were “somewhat” or “not at all confident.” Individuals who reported having no sexual partners were excluded from analyses regarding condom usage in the past 12 months. Odds ratios adjusted for age and gender were calculated to identify whether certain independent variables (Table 1), including condom use problems (Table 3), were associated with confidence in condom use. Adjustment for age was entered into the model as a continuous variable. Where limited by small numbers, variables were regrouped to assist statistical analysis (Table 1). Age, number of standard drinks, and lifetime sexual partners were regrouped as dichotomous variables according to the median (age, <21 versus ≥21; number of drinks, <10 versus ≥10; lifetime sexual partners, <5 versus ≥5). Sexuality was grouped as heterosexual or nonheterosexual (homosexual, bisexual, and other).

## 3. Results

### 3.1. Demographics

Of the 288 participants included for data analysis, 55% were female and 60% were not in a relationship. The median age was 21 years (IQR 19–23). Of all the participants, 89% identified as heterosexual with the remaining identifying as homosexual, bisexual, or other (4%, 5%, and 1%, resp.). Only 6% of those surveyed had never been sexually active. The sample had representation from most Australian states with the majority residing in Queensland (44%) and New South Wales/ACT (40%) and smaller numbers from Victoria, South Australia, and Western Australia.

### 3.2. Confidence

Overall, participants were confident in their ability to use a condom correctly, with 78% responding as either “extremely” or “mostly confident.” Less than one-quarter (23%) of the participants described themselves as “somewhat confident” or “not at all” confident.

Table 1 displays the association between demographic characteristics and risk behaviours with confidence. Confidence was associated with being male (*P* < 0.001), with 43% of males describing themselves as “extremely confident” in using condoms, compared with 14% of females. Conversely, more female participants said they were “not at all” or “somewhat” confident (33%) when compared with males (9%). Additionally, participants aged 21 or older were more confident than those under the age of 21 (*P* = 0.026). Of those who had never been sexually active, 62% (*n* = 8) described themselves as not confident using a condom. Similarly, only one of the participants who had one sexual partner or less (*n* = 40) reported being “extremely confident.” In comparison, 86% (*n* = 44) of those with more than 10 lifetime sexual partners responded as being* either* “mostly confident” or “extremely confident.” Furthermore, 94% (*n* = 15) of participants with more than 20 lifetime sexual partners were “mostly” or “extremely confident” in their ability. Those with greater than five lifetime sexual partners were shown to be two times more likely to be confident than those with less than or equal to five sexual partners (OR 2.13 (1.04–4.37); *P* = 0.038).

Finally, when adjusting for age and gender, those responders who had consumed ten or more standard drinks in the 24-hour period preceding the survey were more than twice as likely to be confident than those who had consumed less than ten (OR 2.40 (1.11–5.15); *P* = 0.025). There was no significant association between sexuality or relationship status and confidence.

### 3.3. Source of Information about Condoms

When asked about how they learnt to put on a condom, 55% of participants selected high school sex education and 27% learnt from a partner, 18% from packet instructions, 17% from either friends or family, 7% from the internet, and 5% from a health-care worker. There were 40 responders (14%) who selected the “other” option, most often using free text to state that they had never learnt to put on a condom correctly or did not know how. High school sex education was not found to be predictive of confidence (*P* = 0.45); however learning from packet instructions was predictive of higher confidence (*P* = 0.011). No other sources of information were associated with higher confidence.

### 3.4. Condom Use Behaviours (Table 2)

The majority found it “easy” to find condoms that fit properly (67%), wear condoms from start to finish (61%), and keep condoms from drying out (63%). When asked about their condom use in the past 12 months, only 18% of respondents said they always used condoms during sex, whilst 78% said they never used a condom for oral sex. The majority (94%) had been under the influence of drugs or alcohol during sex some time in the last year. Approximately one in five (19%) reported being under the influence “most of the time” or “always” when they had sex.

### 3.5. Condom Use Errors

Many participants had experienced difficulties or problems using condoms in the past year (Table 2). Of those who had used a condom in the past year (90%; *n* = 245), 37% had experienced the condom breaking, 48% had experienced the condom slipping off during intercourse, and 51% had experienced the condom slipping off when withdrawing the penis after sex. Interestingly, the majority of participants stated that it would be “easy” for them to keep a condom on when withdrawing after sex (77%) or from breaking during intercourse (60%) (Table 3). When adjusting for age and gender those participants who never checked the expiry date (*n* = 126, 47%) were almost half as likely to be confident than those who sometimes or always checked the expiry date (OR = 0.54 (0.29–1.00); *P* = 0.049) (Table 3). There were no other condom use errors that were associated with confidence.

## 4. Discussion

Overall, 77% of participants were found to be “mostly” or “extremely confident” in their ability to use condoms correctly. This result is encouraging due to the association of confidence (or self-efficacy) with greater condom use amongst young people [9–17]. This population (18–29-year-olds) was chosen due to their relatively increased risk of sexually transmitted infections [2]. The Royal Australian College of General Practitioners (RACGP) recommends targeted screening for STIs in 15–29-year-olds for that reason [24]; however due to ethical considerations people under the age of 18 years were excluded.

Males were found to be significantly more confident than females. This correlates with previous evidence which demonstrates higher self-efficacy in condom use amongst men [25]. Numerous studies have investigated the influence of social attitudes and expected gender roles with respect to condom use [25–27]. Research from 2008 demonstrated that women in traditional relationships had less influence over condom use decisions [26] and those women with more conventional ideas about gender roles were less likely to use a condom [27]. These factors may partially account for women's reduced confidence. A 2014 US study [27] on gender roles and the existence of a sexual double standard describes a wide variation of attitudes and perceptions amongst women regarding condom use. For instance, some women may consider condom application to be the man's responsibility, whilst other women feel that condom application is the duty of both genders. Although our data demonstrates lower reported condom use confidence amongst women, further investigation of this finding is required amongst Australian women to investigate how gender roles may influence condom use behaviours.

Both older age (>21 years) and greater number of lifetime sexual partners (>5) were positively associated with condom use confidence. Importantly, this relationship between confidence and greater number of sexual partners held when adjusted for age. It must be noted that only participants' total number of sexual partners was measured, not sexual encounters. A greater number of sexual encounters would be expected to lead to higher confidence levels; however this would have been largely impractical to measure accurately. Conversely, education avenues such as high school sex education, health care workers, family, friends, and partners were not associated with higher confidence. These findings suggest that confidence may be gained through sexual experience rather than sources of education. Interpretation of this result is somewhat limited by small numbers in each education category, and targeted research would be needed to better explore this finding.

It is of interest that high school sexual education did not demonstrate an association with condom use confidence. High school education is logistically well situated to educate large volumes of young Australians and provide them with information on safe sex practices. Despite high school remaining the primary form of sexual education nationwide [28], 45% of participants did not acknowledge receiving condom use information at high school. Whilst this may have been a shortcoming of the instrument, it could indicate that Australian high school sexual education is not reaching this group or not providing enough information about condom application. Although our results suggest high school sex education does not improve condom use confidence, overseas literature supports its efficacy. A meta-analysis comparing students participating or not participating in school-based sex education showed those who received education had significantly greater self-efficacy relating to condom use, more frequent condom use, and fewer sexual partners (*P* < 0.001) [29]. Contrary to the findings of our study, the result of this meta-analysis suggests that condom efficacy can be improved independently of sexual experience, which is promising for high school educational intervention. Further support comes from a study by Weinstein et al. [30], concluding that greater knowledge of sexual health domains, including contraception and STIs, is associated with greater confidence in condom use. The study also demonstrated that education increased condom use assertiveness amongst females, suggesting that educational interventions may also address some of the barriers to condom use associated with traditional gender roles. Our results may differ due to variations in the high school sexual education curriculum between Australia and other countries and cultural differences or may be due to characteristics of our sample population.

In contrast to high school sexual education, those who had learnt from packet instructions were found to have a significantly greater rating of confidence than those who had not (*P* = 0.011). It is unclear whether this relationship has developed from already confident individuals who are proactive about their sexual health education. Literature exists to suggest the opposite. In a study by Lindemann et al. (2012) [31], those who read the packet instructions did not perform better in a condom application task than those who did not read the instructions. The authors concluded that whilst packet instructions are beneficial for condom application skills, they are not a sufficient method alone for teaching these skills. The results regarding behaviours of the music festival population are consistent with previous evidence that they are a group at high risk of unsafe sexual practices [16, 17]. The majority (94%) of respondents reported being under the influence of drugs or alcohol during sex at some point in the past 12 months. The significance of this arises from findings of a 2011 systematic review which concluded that alcohol consumption was an independent risk factor for intent to engage in unprotected sex [32]. D'Anna et al. [33] found that sex whilst under the influence of drugs or alcohol was the single most significant predictor for condom use problems amongst those attending an STI clinic. Furthermore, our sample reported a high level of recent alcohol consumption, with 34% of participants consuming 10 or more standard drinks in the past 24 hours. Despite the relationship between alcohol and sexual risk, participants who had more than or equal to ten drinks in the last 24 hours, independent of age and gender, were more than twice as likely to be confident in using a condom (OR 2.40 (1.11–5.15); *P* = 0.025). These findings are supported by a 2014 study [34] which shows that alcohol intoxication strengthened condom use self-efficacy amongst a similarly aged group of young women and increased intention to negotiate condom use. However, both the current and aforementioned studies are limited by the use of self-reported measures, and these results may not reflect actual behaviours. The methodological exclusion of those who were visibly intoxicated mitigates but does not nullify the influence that alcohol may have had on survey performance and confidence.

A high number of participants had negative experiences with condoms in the past year, with 37% experiencing a condom breaking and 48% experiencing the condom slipping off during intercourse and 51% when withdrawing the penis after sex. These numbers are considerably higher than figures from previous research on the Australian male population. De Visser et al. [35] in 2003 conducted telephone interviews of 10,173 16–59-year-old Australian men, finding that 23.8% had experienced at least one condom breakage in the past year and 18.1% had experienced at least one condom slippage over the same time. The higher incidence of failures found in festival attendees may in part be explained by younger age of participants and subsequent likelihood to engage in high risk sexual behaviours [33, 35]. Problems with condom use has not yet been investigated in this population, and to our knowledge this is the first study to demonstrate that young Australian festival attendees may be experiencing significantly higher rates of problems when using condoms. Condom errors and failures are established as a significant contributor to STIs and unwanted pregnancy [36–38] and targeting future interventions toward this group may be of value.

This was a cross-sectional study limited by a small sample size and convenience sampling, and greater recruitment of a more diverse population may have increased the power and applicability of the results. Self-selection bias is a further consideration, as participants became involved through their own volition. It is possible that those willing to participate in sexual health research are more interested in their sexual wellbeing or more sexually confident than those who choose not to. Additionally, the use of self-reporting exposes the study to various biases such as participant reactivity and social desirability bias. Respondents were asked if they were currently single, married, or in a relationship, providing current demographic information but no further information about their relationship status over the past year. Whilst the majority of those surveyed (82%) did not always use condoms in the past 12 months, this study does not determine whether that was with single or multiple sexual partners.

It is unclear to what extent the festival sample represents the wider Australian youth, necessitating conservative interpretation of the results. Regardless, the findings may be representative of young Australian music festival attendees, an established at-risk population.

## 5. Conclusion

This study provides a snapshot into the patterns of condom use among young Australian music festival attendees. Despite reasonable levels of confidence in their ability to use condoms, inconsistent use and a high rate of condom failure put this population at an increased risk of sexually transmitted infections. Such results reflect the need for targeted intervention in the young music festival attendee population. For many years, the focus of sexual health literature has been on attitudes toward condoms and frequency of condom use. This study adds to a growing body of evidence that highlights the association between perceived self-efficacy or confidence using condoms and incorrect use of condoms causing errors. It also exposes the need to better understand whether higher confidence or self-efficacy is predictive of real-world condom application skills and intention to use condoms. Ultimately, this study has implications for sexual health promotion and public health programs by demonstrating the need to empower young Australians, particularly females, with knowledge and confidence in their own ability to use condoms.

## Figures and Tables

**(a):** 

Variable	All		Confident					Odds ratio	
			Yes		No		*P* value	Adjusted odds ratio ^*∗*^ (95% CI)	*P* value: adjusted odds ratio ^*∗*^
			Extremely	Mostly	Some what	Not at all			
	*n*	%	*n* (%)		*n* (%)				
Gender	288						<**0.001** ^a^		<**0.001**
Male	129	45	55 (43)	62 (48)	12 (9)	0 (0)		4.70 (2.37–9.31)	
Female	159	55	22 (14)	84 (53)	42 (26)	11 (7)		1	
Age	288						0.026^a^		0.386
<21	139	48	26 (19)	76 (55)	31 (22)	6 (4)		1	
≥21	149	52	51 (34)	70 (47)	23 (15)	5 (3)		1.29 (0.72–2.32)	
Relationship status	288						0.173^a^		0.222
Married/in a relationship	114	40	93 (82)		21 (18)			1.46 (0.80–2.69)	
Single	174	60	130 (75)		44 (25)			1	
Sexuality	280						0.204^ab^		0.187^b^
Heterosexual	249	89	192 (77)		57 (23)			1	
Homosexual	12	4	10 (83)		2 (17)			2.13 (0.69–6.59)	
Bisexual	15	5	14 (93)		1 (7)				
Other	4	1	3 (75)		1 (25)				
Number of drinks in the last 24 hours	271						0.009^f^		0.025^f^
0	17	6	15 (88)		2 (12)			1	
1–5	74	27	50 (68)		24 (32)				
6–9	89	33	65 (73)		24 (27)				
10–20	69	26	62 (90)		7 (10)			2.40 (1.11–5.15)	
>20	22	8	19 (86)		3 (14)				
Number of lifetime sexual partners	237						0.007^g^		0.038^g^
0	13	6	1 (8)	4 (31)	5 (39)	3 (23)			
1	27	11	0 (0)	19 (70)	5 (19)	3 (11)			
2–5	86	36	25 (29)	41 (48)	18 (21)	2 (2)			
6–10	60	25	18 (30)	34 (57)	7 (12)	1 (2)			
11–20	35	15	12 (34)	17 (49)	5 (14)	1 (3)			
>20	16	7	11 (69)	4 (25)	1 (6)	0 (0)			
≤5	126	53	26 (21)	64 (51)	28 (22)	8 (6)		**1**	
>5	111	47	41 (37)	55 (50)	13 (12)	2 (2)		**2.13 (1.04–4.37)**	

**(b):** 

Source of education	All (*n*)	All (%)	Confident, *n* (%)	Not confident, *n* (%)	*P* value
High school	158	55	125 (79)	33 (21)	0.451^a^
Partner	77	27	60 (78)	17 (22)	0.904^a^
Packet instructions	53	18	48 (91)	5 (9)	0.011^a^
Friends	40	14	31 (78)	9 (23)	0.991^a^
Other	40	14	32 (80)	8 (20)	0.675^a^
Internet	19	7	18 (95)	1 (5)	0.085^c^
Health care workers	15	5	14 (93)	1 (7)	0.204^c^
Family	9	3	9 (100)	0 (0)	0.216^c^

**(c):** 

Variable	Median (IQR), all participants	Median (IQR), confident	Median (IQR), not confident	*P* value
Age	21 (19–23)	21 (19–23)	20 (19–22)	0.089^e^
Number of standard drinks, past 24 hours	10 (4–15)	10 (5–15)	6 (3–10)	0.005^e^
Number of sexual partners, lifetime	5 (2–10)	6 (3–10)	4 (1–7)	0.001^e^

a: Chi-square test; b: *P* value: heterosexual versus nonheterosexual; c: Fisher's exact test; d: interquartile range; e: Mann-Whitney *U* test; f: *P* value: <10 drinks versus ≥10 drinks; g: *P* value: ≤5 versus >5 sexual partners. ^*∗*^Adjusted for age and gender.

**(a):** 

	Difficult (1-2)			Neutral (3)			Easy (4-5)		
How easy or difficult would it be to:	Male *n* (%)	Female *n* (%)	Total *n* (%)	Male *n* (%)	Female *n* (%)	Total *n* (%)	Male *n* (%)	Female *n* (%)	Total *n* (%)
Find condoms that fit properly (*n* = 233)	4 (3)	11 (10)	**15 (6)**	25 (20)	36 (34)	**61 (26)**	98 (77)	59 (56)	**157 (67)**
Put a condom on correctly (*n* = 280)	0 (0)	1 (1)	**1 (0.4)**	58 (46)	115 (75)	**173 (62)**	68 (54)	38 (25)	**106 (38)**
Keep a condom from drying out during sex (*n* = 272)	11 (9)	25 (17)	**36 (13)**	26 (21)	38 (26)	**64 (24)**	86 (70)	86 (58)	**172 (63)**
Keep a condom from breaking during sex (*n* = 271)	11 (8)	19 (13)	**30 (11)**	36 (29)	42 (29)	**78 (29)**	79 (63)	84 (58)	**163 (60)**
Keep an erection while using a condom (males: *n* = 127)	14 (11)			24 (19)			89 (70)		
Keep a condom on when withdrawing after sex (males: *n* = 125)	5 (4)			24 (19)			96 (77)		
Wear a condom from start to finish during sex (males: *n* = 126)	23 (18)			26 (21)			77 (61)		

**(b):** 

Behaviour amongst those who have had sex	Never (1)			Sometimes (2–4)			Always (5)		
In the last 12 months, how often have you:	Male *n* (%)	Female *n* (%)	Total *n* (%)	Male *n* (%)	Female *n* (%)	Total *n* (%)	Male *n* (%)	Female *n* (%)	Total *n* (%)
Used a condom during sex (*n* = 272)	14 (11)	13 (9)	**27 (10)**	93 (76)	102 (69)	**195 (72)**	16 (13)	34 (23)	**50 (18)**
Used a condom during oral sex (*n* = 272)	98 (80)	113 (75)	**211 (78)**	23 (19)	33 (22)	**56 (21)**	1 (1)	4 (3)	**5 (2)**
Been under the influence of alcohol or other drugs during sex (*n* = 270)	9 (7)	7 (5)	**16 (6)**	108 (88)	135 (92)	**243 (90)**	6 (5)	5 (3)	**11 (4)**

**(c):** 

Behaviour amongst those who have used condom in the last 12 months	Never (1)			Sometimes (2–4)			Always (5)		
In the last 12 months, how often have you:	Male *n* (%)	Female *n* (%)	Total *n* (%)	Male *n* (%)	Female *n* (%)	Total *n* (%)	Male *n* (%)	Female *n* (%)	Total *n* (%)
Experienced the condom breaking during intercourse (*n* = 268)	66 (55)	102 (70)	**168 (63)**	55 (45)	45 (31)	**100 (37)**	0 (0)	0 (0)	**0 (0)**
Experienced the condom slipping off during intercourse (*n* = 270)	60 (49)	81 (55)	**141 (52)**	63 (41)	66 (45)	**129 (48)**	0 (0)	0 (0)	**0 (0)**
Experienced the condom slipping off when removing it after intercourse (*n* = 270)	60 (49)	72 (49)	**132 (49)**	61 (50)	76 (51)	**137 (51)**	1 (1)	0 (0)	**1 (0.4)**
Checked the expiry date on the condom packet (*n* = 271)	57 (47)	69 (46)	**126 (46)**	55 (45)	62 (42)	**117 (43)**	10 (8)	18 (12)	**28 (10)**
Used oil-based lubricant (baby oil, vaseline) (*n* = 272)	71 (58)	89 (60)	**160 (59)**	51 (42)	58 (39)	**109 (40)**	1 (1)	2 (1)	**3 (1)**

**Table 3:** Condom use errors as predictors of confidence.

Variable error	All			Confidence				Odds ratio			
	Male *n* (%)	Female *n* (%)	Total *n* (%)	Yes *n* (%)		No *n* (%)		Unadjusted odds ratio (95% CI)		Adjusted odd ratio^*∗*^(95% CI)	*P* value Adjusted odds ratio^*∗*^
				Male *n* (%)	Female *n* (%)	Male *n* (%)	Female *n* (%)	Male	Female		
Condom breaking in the last 12 months (*n* = 268)											0.677
Never	67 (40)	101 (60)	**168 (63)**	61 (46)	73 (55)	6 (18)	28 (82)	0.80 (0.21–2.98)	1.30 (0.61–2.78)	1	
Sporadic/always	55 (55)	45 (45)	**100 (37)**	51 (63)	30 (37)	4 (21)	15 (79)	1	1	1.15 (0.60–2.22)	

Condom slipping off during intercourse in last 12 months (*n* = 269)											0.436
Never	60 (43)	80 (57)	**140 (52)**	56 (50)	57 (50)	4 (15)	23 (85)	1.75 (0.49–6.31)	1.16 (0.57–2.35)	1.28 (0.69–2.38)	
Sporadic/always	63 (49)	66 (51)	**129 (48)**	56 (55)	45 (44)	7 (25)	21 (75)	1	1	1	

Condom slipping off when withdrawing in last 12 months (*n* = 269)											0.252
Never	60 (46)	71 (54)	**131 (49)**	56 (52)	52 (48)	4 (17)	19 (83)	1.78 (0.49–6.43)	1.34 (0.66–2.73)	1.44 (0.77–2.67)	
Sporadic/always	62 (45)	76 (55)	**138 (51)**	55 (52)	51 (48)	7 (22)	25 (78)	1	1	1	

Checking expiry date in last 12 months (*n* = 270)											**0.049**
Never	57 (45)	69 (55)	**126 (47)**	49 (52)	45 (48)	8 (25)	24 (75)	0.30 (0.08–1.18)	0.64 (0.31–1.29)	0.54 (0.29–1.00)	
Sporadic/always	65 (55)	79 (45)	**144 (53)**	62 (51)	59 (49)	3 (13)	20 (87)	1	1	1	

Used oil-based Lubricant in last 12 months (*n* = 270)											0.937
Never	71 (44)	89 (56)	**160 (59)**	65 (51)	62 (49)	6 (18)	27 (82)	1.15 (0.33–4.00)	0.93 (0.45–1.91)	0.98 (0.52–1.83)	
Sporadic/always	52 (46)	59 (54)	**111 (41)**	47 (53)	42 (47)	5 (23)	17 (77)	1	1	1	

^*∗*^Adjusted for age and gender, eliminating all nonresponders and nonusers of condoms.
